# Randomised, single-masked non-inferiority trial of femtosecond laser-assisted versus manual phacoemulsification cataract surgery for adults with visually significant cataract: the FACT trial protocol

**DOI:** 10.1136/bmjopen-2015-010381

**Published:** 2015-11-27

**Authors:** Alexander C Day, Jennifer M Burr, Catey Bunce, Caroline J Doré, Yvonne Sylvestre, Richard P L Wormald, Jeff Round, Victoria McCudden, Gary Rubin, Mark R Wilkins

**Affiliations:** 1 UCL Institute of Ophthalmology, University College London, London, UK; 2 The NIHR Biomedical Research Centre at Moorfields Eye Hospital NHS Foundation Trust and UCL Institute of Ophthalmology, London, UK; 3 University of St Andrews, Scotland, Fife, UK; 4 UCL Comprehensive Clinical Trials Unit, London, UK

**Keywords:** Laser assisted cataract surgery, Phacoemulsification cataract surgery, Randomised controlled trial

## Abstract

**Introduction:**

Cataract is one of the leading causes of low vision in the westernised world, and cataract surgery is one of the most commonly performed operations. Laser platforms for cataract surgery are now available, the anticipated advantages of which are broad and may include better visual outcomes through greater precision and reproducibility, and improved safety. FACT is a randomised single masked non-inferiority trial to establish whether laser-assisted cataract surgery is as good as or better than standard manual phacoemulsification.

**Methods and analysis:**

808 patients aged 18 years and over with visually significant cataract will be randomised to manual phacoemulsification cataract surgery (standard care) or laser-assisted cataract surgery (intervention arm). Outcomes will be measured at 3 and 12 months after surgery. The primary clinical outcome is uncorrected distance visual acuity (UDVA, logMAR) at 3 months in the study eye recorded by an observer masked to the trial group. Secondary outcomes include UDVA at 12 months, corrected distance visual acuity at 3 and 12 months, complications, endothelial cell loss, patient-reported outcome measures and a health economic analysis conforming to National Institute for Health and Care Excellence standards.

**Ethics and dissemination:**

Research Ethics Committee Approval was obtained on 6 February 2015, ref: 14/LO/1937. Current protocol: v2.0 (08/04/2015). Study findings will be published in peer-reviewed journals.

**Trial registration number:**

ISRCTN: 77602616.

**Strengths and limitations of this study**

- This is the first randomised multicentre UK trial of laser-assisted cataract surgery compared to manual phacoemulsification cataract surgery, recruiting 808 patients over 18 months with a 12-month follow-up period.
- Outcome measures include visual outcomes, complications, visual function and quality of life measurements.
- The primary outcome measure is uncorrected distance visual acuity at 3 months postoperatively and will be recorded by masked optometrists.
- The trial findings will help inform National Health Service providers, commissioners, patients and ophthalmologists about the effectiveness and cost-effectiveness of these two methods of cataract surgery.
- A high recruitment rate will be required for the trial to complete on schedule.

## Introduction

Cataract is the leading cause of blindness in the world,^1^ and one of the leading causes of low vision in the UK.^2^ Cataract surgery is the most commonly performed operation by the UK National Health Service (NHS) with around 330 000 cataract operations performed per year in England.^3^ The current surgical method used for over 99%^4^ cases was first introduced into routine clinical practice over 20 years ago and uses ultrasound (phacoemulsification) to help break up the cataract.

After cataract surgery, for eyes without ocular copathology, 51% and 95% achieve visual acuities of 0.00 and 0.30 logMAR respectively;^3^ which are those perceived as normal vision, and the minimum standard for driving in the UK. Although cataract surgery is a relatively safe operation, serious complications that may affect recovery and are associated with poorer long-term outcomes occur in 2% operations.^3^ Posterior capsule rupture/vitreous loss (PCR/VL) is the most frequently occurring serious complication and is associated with an eight times higher risk of postoperative endophthalmitis and a 42 times higher risk of undergoing retinal detachment surgery within 3 months.^3^ The surgical learning curve for phacoemulsification is associated with complications, with a 3.7 and 1.6 times higher risk of PCR/VL for junior surgical trainees and senior surgical trainees, respectively.^5^ In cases with PCR/VL, one-third of patients still have symptoms about their eye and vision 3.5 years after surgery.^6^

Laser platforms for cataract surgery are currently available from five manufacturers. They can make the corneal incisions, open the lens capsule and fragment the cataract in 1 min, leaving only removal of lens fragments and insertion of the lens implant to be performed by the surgeon. The potential advantages are broad and may include better visual outcomes through greater precision and improved safety. These systems are expensive, however costs may be mitigated by fewer complications and improved outcomes. The absence of robust evidence supporting the safety and efficacy of laser-assisted surgery from large randomised controlled trials was highlighted in a review article^7^ and by the National Institute of Health Research Horizon Scanning Centre. The topic is a research priority as identified by the national James Lind Alliance Sight Loss and Vision Priority Setting Partnership (see http://www.fightforsight.org.uk/sightlosspsp). A 1050 patient multicentre randomised economic evaluation is currently underway in France with planned primary completion in July 2015.^8^ To date, data from large comparative case series suggest visual outcomes from laser cataract surgery are similar to, or possibly better than, those from manual phacoemulsification.^9^ ^10^

### Trial objectives and design

The aim of this multicentre, single-masked randomised controlled non-inferiority trial is to establish whether laser-assisted cataract surgery is as good as or better than standard surgery. The trial will help inform NHS service providers, commissioners, patients and ophthalmologists about the effectiveness and cost-effectiveness of these two methods of cataract surgery.

The hypotheses being tested are that those randomised to laser-assisted cataract surgery will have postoperative visual acuities (uncorrected and corrected distance visual acuity, UDVA and CDVA respectively) as good as or better than those randomised to standard care and improvements in endothelial cell loss, self-reported visual function (assessed by the Catquest-SF9 questionnaire^11^) and EQ-5D quality of life score.^12^

*Population*: 808 patients with visually symptomatic cataract (404 per arm).

*Intervention*: Laser-assisted cataract surgery.

*Control*: Standard phacoemulsification cataract surgery.

*Outcomes*: visual acuity, visual function, refractive outcomes, complications including endothelial cell loss and cost-effectiveness.

## Methods

### Study setting

The study sites are high volume NHS day care surgery units that see large numbers of patients for routine cataract surgeries and therefore have a sufficiently large pool of patients to recruit successfully to a large randomised study. These sites have access to the laser equipment and offer a realistic NHS scenario.

### Eligibility criteria

#### Inclusion criteria

Patients must meet all of the following criteria at the time of randomisation to be eligible for recruitment:

- Adult aged 18 or over with visual symptoms attributed by the examining ophthalmologist to the presence of cataract in one or both eyes.
- Willing to attend for follow-up 3 and 12 months after cataract surgery in the first eye.
- Sufficiently fluent in English for informed consent and self-completion of the health state questionnaires.
- The postoperative intended refractive target in the study eye is within ±0.5 dioptres of emmetropia (ie, the postoperative refractive target is good distance vision).

The study eye is defined as the first eye to undergo cataract surgery in the trial. This is chosen by the patient in discussion with the surgeon. In those requiring bilateral cataract surgery, the same intervention will be offered for both eyes unless the patient wishes otherwise.

In addition to the above, trial exclusion criteria include any of the following:

- Corneal, ring and/or inlay implants, severe corneal opacities, corneal abnormalities, significant corneal oedema and diminished aqueous clarity that is likely to obstruct the optical coherence tomography imaging of the anterior lens.^i^
- Descemetocele with impending corneal rupture.^i^
- Subluxed crystalline lens.^i^
- Poor pupil dilation that is expected to require surgical iris manipulation.
- Patient unable to give consent or attend follow-up assessments.
- Patient unable to be positioned for surgery.^i^
- Scheduled for combined surgery, for example, cataract and trabeculectomy.
- Any clinical condition that the investigator considers would make the patient unsuitable to take part in the trial.

### Interventions

#### Standard care/control arm: manual phacoemulsification cataract surgery

This is the standard method of cataract surgery at participating trial sites.

Typically this will involve topical or local anaesthesia (with or without concurrent sedation depending on patient or surgeon preference). The eye to undergo cataract surgery will be dilated according to standard local unit practice.

Following local standard surgical checks (WHO guidelines), the patient will be randomised before being transferred to the anaesthetic room or direct to the operating theatre where the intended anaesthetic is given.

Phacoemulsification cataract surgery will then be performed according to local standard practice and the study operating manual. A plan for astigmatism will be made prior to randomisation. Postoperative care including postoperative eye drops will be as per standard unit practice for cataract surgery.

The surgery start time will be defined as application of antiseptic solution to the periocular region by the operating surgeon following the final patient check. The surgery end time will be defined as the time of removal of the surgical drape.

### Intervention arm: laser-assisted cataract surgery

Patients undergoing laser-assisted cataract surgery will be prepared for surgery in the same way as those in the standard care arm.

The patient will be transferred to the laser room prior to the operating theatre, or to the operating theatre if the laser platform is sited there. If a depot pellet has been used for dilation, this will be removed at this stage. Anaesthetic eye drops will be administered, the laser interface placed in contact with the eye with the patient lying down, the interface is docked to the laser platform, the eye is scanned by the integral guidance and the laser treatment delivered. The laser docking and treatment delivery will be in accordance with the laser manufacturer's recommended procedure as detailed in the relevant operating manual.

The laser will be used to perform capsulotomy (typically 4.8–5.5 mm diameter), lens fragmentation and corneal incisions including astigmatic keratotomies. A recommended nomogram will be provided for planning of the astigmatic keratotomies.

Following laser delivery, additional dilating drops will be administered. The patient will be transferred to the operating theatre if applicable and the remainder of the care pathway is as per standard care with the obvious exception that the surgical steps completed by laser do not need to be performed by the surgeon.

Where the laser treatment cannot be performed for whatever reason following randomisation to laser-assisted cataract surgery (eg, unable to dock, laser machine fault, etc) patients will undergo surgery in accordance to that for standard care.

### Astigmatism correction

All surgeons will be asked to describe their planned method of attempted astigmatism correction prior to surgery (including femtosecond laser astigmatic keratotomy for those randomised to laser).

### Surgeon eligibility

Any ophthalmologists who routinely perform cataract surgery at their respective trial sites and who have completed at least 10 supervised laser-assisted cataract surgery operations will be able to perform surgery for the laser-assisted or standard phacoemulsification trial arms.

### Outcome measures

#### Primary outcome

- Uncorrected distance visual acuity (UDVA, logMAR) at 3 months following surgery in the study eye measured using a standard Early Treatment Diabetic Retinopathy Study (ETDRS) chart at a starting distance of 4 m.

#### Secondary outcomes

- UDVA at 12 months.
- CDVA, logMAR at 3 and 12 months in the study eye (ETDRS logMAR chart at a starting distance of 4 m).
- Ocular complications within 3 and 12 months in the study eye. A complication will be defined as any event that causes unintentional injury to an ocular structure, or requires additional treatment, or has a negative effect on a patient’s health or eyesight.
- Uncorrected and corrected visual acuity and complications in the second eye (for those with bilateral cataracts), and with both eyes open at 3 and 12 months (after surgery on the first eye).
- Refractive error (spherical equivalent) within 0.5 and within 1 dioptre of intended refractive outcome for each eye.
- Quality of life as measured by the EQ-5D-3 L questionnaire+vision bolt-on question (EQ-5DV) at 6 weeks, 3, 6 and 12 months.^12^
- Patient-reported vision health status using Catquest–9SF,^11^ a Rasch validated instrument at 6 weeks, 3, 6 and 12 months.
- Cost-utility analysis reported as the incremental cost-effectiveness ratio and cost-effectiveness acceptability curves.
- Corneal endothelial cell count change at 3 and 12 months.

### Participant timeline

**Figure 1:**
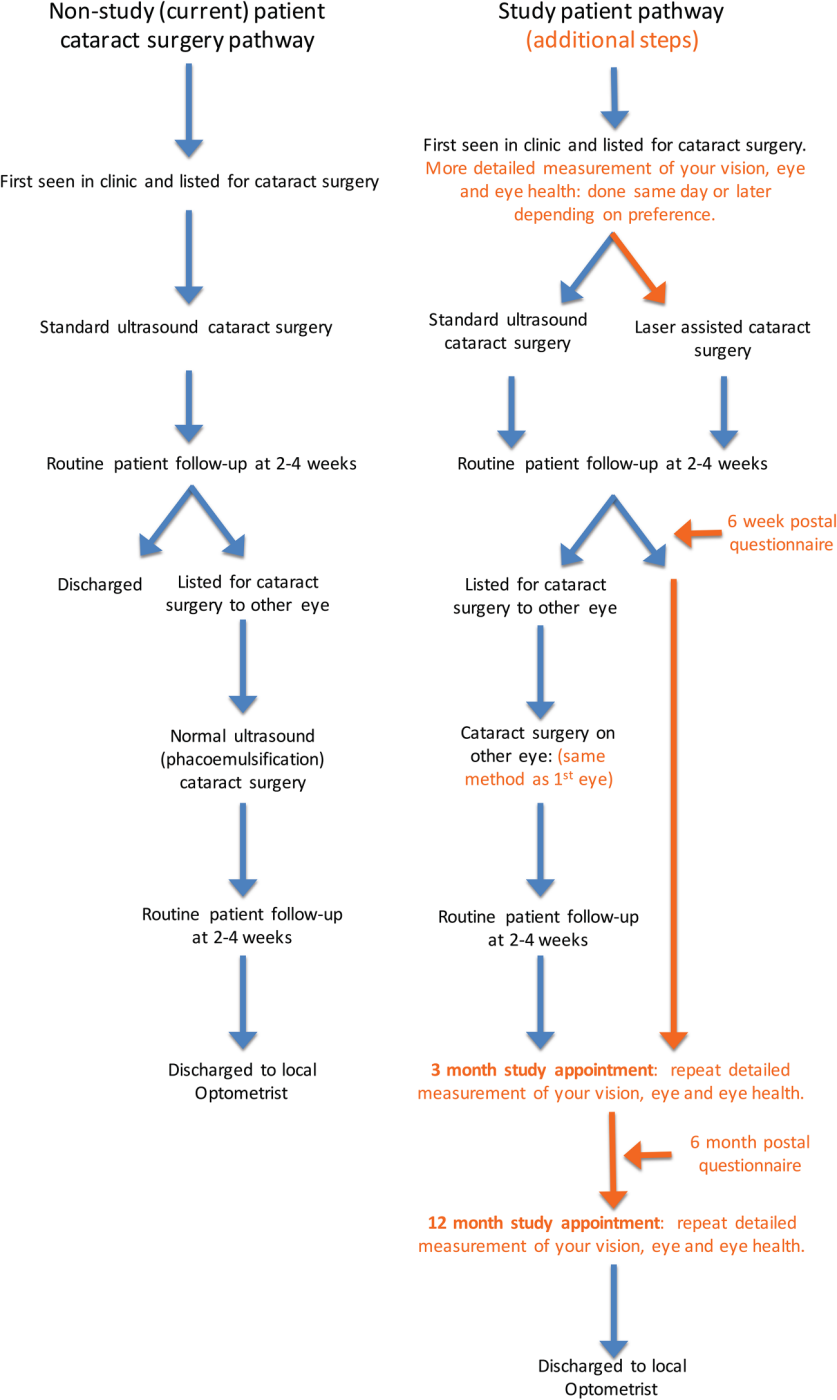
Summary trial participant pathways.

This can been seen in summary trial participant pathways table 1 and figure 1.

**Table 1:** Schedule for data collection and visits

	Normal	Baseline	Allocation	Treatment	Follow-up				
	Prior to enrolment		Prior to surgery	Surgery	Standard non-study postoperative visit	6 weeks (by post)	Visit 1: 3 months	6 months (by post)	Visit 2: 12 months
Medical and ocular history	✓	✓							
Consent for cataract surgery	✓								
Informed consent and eligibility screening		✓							
Identification of study eye		✓							
Visual acuity: UDVA, pinhole, +/− glasses (Snellen)	✓		✓*		✓				
Visual acuity (logMAR) with usual method of correction			✓						
Visual acuity: UDVA† and CDVA‡ (logMAR) each eye and binocular							✓		✓
Subjective refraction							✓		✓
Ocular biometry	✓								
Pentacam corneal topography	✓§	✓					✓		✓
Optical coherence tomography (OCT)	✓§	✓			✓§		✓		✓
Inclusion/exclusion criteria		✓	✓						
Catquest-9SF questionnaire		✓				✓	✓	✓	✓
EQ-5D-3L+vision bolt-on question (EQ-5DV)		✓				✓	✓	✓	✓
CSRI**		✓					✓	✓	✓
Endothelial cell count measurement	✓§	✓					✓		✓
Randomisation			✓						
Treatment: standard care: Manual Phacoemulsification Cataract Surgery				✓					
Treatment: Intervention: Femtosecond Laser-Assisted Phacoemulsification Cataract Surgery				✓					
Adverse event collection				✓		✓††	✓	✓††	✓

*Current glasses or unaided.

†UDVA: uncorrected distance visual acuity. All visual acuity measures will use the standard ETDRS logMAR chart at 4 metres.

‡CDVA: corrected distance visual acuity using subjective refraction result.

§Some patients will have these tests performed at the standard preassessment visit, depending on the site local procedure for surgical preassessment.

**The CSRI is a questionnaire for collecting retrospective information about study patients’ use of health and social care services, accommodation and living situation, income, employment and benefits.^17^

††Patient reported complications only.

CDVA, corrected distance visual acuity; CSRI, Client Service Receipt Inventory; ETDRS, Early Treatment Diabetic Retinopathy Study; UDVA, uncorrected distance visual acuity.

### Trial duration

The study will take 30 months. Patient recruitment will take place over the first 18 months, and collection of the data will continue for a further 12 months. The trial will be considered closed when the last patient has reached this time-point, all data is complete and all data queries have been resolved. The REC will be notified within 90 days of the end of the trial and sent a summary report of the research within 12 months of the end of the trial.

### Sample size

The primary clinical outcome is UDVA (logMAR) at 3 months in the study eye recorded by an optometrist or technician masked to the trial group. A change in visual acuity of 1 line of the chart is considered to be clinically important. One logMAR line is 5 letters (each letter is 0.02 logMAR) and the test-retest variability is reported as about 0.07 logMAR on letter-by-letter scoring.^13^ ^14^ If there is truly no difference in mean logMAR between the two groups, then 432 patients (216 per group) would provide 90% power to be sure that a 95% two-sided CI would exclude the non-inferiority limit of 0.1 logMAR, assuming a common SD of 0.32. The SD is from the Royal College of Ophthalmologists’ National Ophthalmic Database UDVA data.^3^

However, although treatment is delivered on an individual basis, each patient cannot be assumed to generate independent information since they will be clustered within surgeons. To take account of clustering by surgeon (ie, the variation between surgeons in the treatment effect) the sample size must be increased by an inflation factor f=1+(m−1)×p. Assuming a total of 16 surgeons contribute and an average cluster size (m) of 50 (patients/surgeon) and an estimated ICC (p) of 0.012, this yields an f of 1.59. A total of 688 patients (344 per group) would enable the trial to take account of clustering by surgeon. To allow for an anticipated 15% dropout rate (the mean age of patients undergoing cataract surgery is 75 years old and many have significant systemic comorbidities) the total sample size required is 808 patients (404 per group).

### Recruitment plan

All patients presenting to the recruitment centres with visually significant cataract who have elected to undergo cataract surgery will be considered for enrolment. All potential participants will be provided in advance with a copy of the trial participant information sheet (see online supplementary files for model copy of the trial participant information).

Eligible patients will be identified by an ophthalmologist at the time of listing for cataract surgery and if willing to join the study, informed consent will be obtained and physical baseline measurements taken after standard clinic listing and surgical consent for cataract surgery by a trained member of the trial team. All patients will be given as much time as they require and offered separate alternative day enrolment appointments (see online supplementary files for model copy of the trial consent form).

### Randomisation and allocation

Patients will be randomised to laser-assisted cataract surgery or manual phacoemulsification cataract surgery (standard care) in equal proportions. Randomisation will be performed by a member of the trial team on the day of surgery and as close to the time of surgery as possible. A web-based randomisation application will be used (https://www.sealedenvelope.com/uclctu/). The randomisation algorithm will use treatment centre, surgeon and one or both eyes eligible as minimisation stratifiers.

### Masking

Owing to the nature of the intervention, neither the trial participants nor the treating clinician will be masked to the treatment allocation.

To ensure masking of the outcomes data; visual acuity, refraction, corneal measurements and endothelial cell count measures at 3 and 12 months follow-up visits will be performed by a trial optometrist or technician without reference to previous patient medical notes or trial case report forms. After these measures have been completed, complications data will be collected by patient medical notes review, for which masking is not possible. Each site will be responsible for putting into place a procedure to ensure the optometrist is kept masked at all times, and will remind each patient not to reveal their treatment to the optometrist at these two visits.

### Withdrawal

Patients may withdraw at any time. All withdrawals from randomised treatment will be reported. The investigator may withdraw patients from the study in the event of intercurrent illness, AEs, SAEs, protocol violations, administrative or other reasons. All data will be analysed on an intention-to-treat basis.

### Data management

All study data will be managed as detailed in the full trial protocol and in accordance with the UK Data Protection Act 1998. The trial database and coding frames have been developed by the Clinical Trial Manager in conjunction with CCTU. The database software provides a number of features to help maintain data quality, including; maintaining an audit trail, allowing custom validations on all data, allowing users to raise data query requests and search facilities to identify validation failure/ missing data.

Requests for access to trial data will be considered, and approved in writing where appropriate, after formal application to the Trial Management Group/ Trial Steering Group. Considerations for approving access are documented in the Trial Management Group/Trial Steering Committee Terms of Reference.

### Statistical methods

The primary analysis will be conducted following the intention-to-treat principle where all randomised patients are analysed in their allocated group whether or not they receive their randomised treatment. Baseline characteristics will be summarised for each treatment groups. Continuous data will be summarised using means and SDs if data appear Gaussian, or medians and IQRs. Binary data will be reported as frequencies and percentages. All statistical tests will use a two-sided p value of 0.05 unless otherwise specified. All CIs presented will be 95% and two sided. A detailed statistical analysis plan will be developed for approval by the Trial Steering Committee and review by the Independent Data Monitoring Committee and finalised before the first substantive statistical analysis.

The primary outcome is UDVA in the study eye 3 months after randomisation, measured in logMAR using an ETDRS chart at a starting distance of 4 m. A two-sided 95% CI for the mean difference in UDVA between treatment groups will be estimated using regression analysis adjusting for baseline habitual logMAR visual acuity and the randomisation stratifiers (treatment centre, surgeon and whether or not patients have one or both eyes eligible).

We will include trial site in our regression models as a fixed effect and surgeon as a random effect. If the upper end of the 95% CI for the difference between means does not cross the non-inferiority limit of 0.1 logMAR, then laser surgery will be regarded as non-inferior. If the mean difference is negative and its 95% CI lies wholly to the left of zero, then we can conclude that laser surgery is superior to manual surgery. We will perform sequential testing of the non-inferiority and superiority hypotheses.

Secondary continuous outcomes such as UDVA at 12 months, CDVA and patient-reported Catquest-9SF^11^ will be analysed in a similar fashion. The percentage of study eyes (and patients) experiencing adverse events (eg, posterior capsule tears, dropped lens) in the two groups will be compared using Fisher's exact tests.

In addition regression analyses will be performed for continuous outcomes such as CDVA at 12 months for all operated eyes (ie, including the second eye for patients having bilateral surgery), adjusting for baseline habitual logMAR visual acuity, the randomisation stratifiers (centre and surgeon) and time since surgery, including patient as a random effect. No additional research visits are planned for patients having surgery on both eyes.

Pearson's correlation coefficients (or Spearman's rank correlation coefficients, depending on the distribution of the data) will be used to assess the relationships between continuous outcome measures.

Planned subgroup analyses will be conducted to investigate possible interactions between treatment effects and whether or not surgery was required on both eyes, with separate estimates and CIs being reported for such patients. We will also investigate a possible interaction between treatment effect and trial site as a prespecified subgroup analysis.

### Missing data

While there is no planned research visit between surgery and the postoperative visit at 3 months, it is likely that patients will attend standard NHS visits following surgery. If data are missing for the primary end point because patients do not attend for follow-up at 3 months, visual acuity data will be extracted from patient records and inputted for the primary analysis. If after retrieving the NHS records a substantial proportion of primary outcome data remains incomplete, then missing data will be dealt with using multiple imputation by chained equations. Results will be combined using Rubin's rules. Data will be assumed to be missing at random, in essence the data available for patients before they drop out will be used to predict the end point. The imputation will be performed following a prespecified procedure and conducted separately for each trial group. Reasons for missingness may be important and these will be investigated using logistic regression of covariates on an indicator of missingness. Sensitivity analysis will investigate the validity of the missing at random assumption.

### Economic analysis

We will undertake a detailed analysis of the costs and the cost-utility of laser-assisted phacoemulsification cataract surgery compared with manual phacoemulsification cataract surgery (standard care). The analyses will conform to accepted economic evaluation methods (eg, NICE methods guidance). All costs will be assessed from the perspective of the NHS and personal social services. Secondary analysis will consider patient and societal costs. We will estimate cost and cost-utility (1) for the ‘within-trial’ period, based on the clinical and health-related quality of life results at baseline and follow-up and (2) over the expected lifetime of the patients. We have selected the EQ-5D-3L+vision bolt-on question (EQ-5DV)^12^ as the most appropriate instrument for use in this population.

### Within-trial analysis

The units of outcome for the within-trial cost-effectiveness analyses will be the incremental cost per unit change in the uncorrected distance visual acuity (UDVA) in the study eye and the incremental cost per quality-adjusted life year (QALY) gained. QALYs will be calculated based on the responses to the EQ-5DV collected at baseline and follow-up. Patient-specific utility profiles will be constructed assuming a linear change in utility values measured using the EQ-5DV questionnaire at baseline and 6-week, 3-month, 6-month and 12 month follow-up time points. Utility estimates will be calculated according to the area under the curve approach, adjusting for baseline differences in patients in the trial arms if necessary. Missing EQ-5DV and resource use data will be addressed using appropriate statistical methods in consultation with the trial statistician.

### Model-based analysis

In the lifetime model, cost-effectiveness will be calculated in terms of the incremental cost per QALY gained. A review of previous cost-effectiveness and cost-utility analyses will be conducted to identify any existing modelling work that may be drawn on for developing the model structure and informing model parameters. The specific details of the data required to populate the model will be determined following the development of the model structure. We will undertake deterministic (one, two and multiway) and probabilistic sensitivity analysis, the latter assuming appropriate distributions and parameter values that will also be used to construct cost-effectiveness acceptability curves.

### Health economic analysis plan

All analyses will be undertaken within a Bayesian framework. Methods for conducting economic evaluation using clinical trial data will be applied following O'Hagan and Stevens^15^ and O'Hagan *et al*.^16^ Monte Carlo simulation methods will be used to construct a cost-effectiveness acceptability curve, based on the expected net benefit statistic, to estimate the probability that the intervention is cost-effective for a range of values of societal willingness to pay per QALY. We will also subject the results to extensive deterministic (one, two and multiway) sensitivity analysis.

Cost components included in the analysis will consist of (but not necessarily be limited to) the cost of surgery for both arms of the trial, complications arising from surgery, all relevant diagnostic investigations, revision surgery where necessary, hospital length of stay, outpatient attendances, hospital readmissions, primary care contacts, A&E attendances and prescribed medications. The volume of resource use for each cost component will be measured by a patient completed questionnaire and from medical records. Patients will be asked for details of any health and social care resources used during the study period, as well as out of pocket payments and the impact of their condition on their employment. Unit costs will be taken from standard published sources where possible. Where published unit costs are not available site specific unit costs will be obtained as required.

### Trial oversight

Trial management team (TMT): this will assist with developing the design, co-ordination and day-to-day operational issues in the management of the trial, including budget management.

Trial management group (TMG): this will assist with developing the design, co-ordination and strategic management of the trial. The membership, frequency of meetings, activity (including trial conduct and data review) and authority will be covered in the TMG terms of reference.

Independent Trial Steering Committee (TSC): is the independent group responsible for oversight of the trial in order to safeguard the interests of trial participants. The TSC provides advice to the CI, CCTU, the funder and sponsor on all aspects of the trial through its independent Chair. The membership, frequency of meetings, activity (including trial conduct and data review) and authority will be covered in the UCL CCTU TSC terms of reference.

Independent Data Monitoring Committee (IDMC): will monitor adverse events and serious adverse events during the trial to inform their recommendations to the TSC. The IDMC will be responsible for safeguarding the interests of all trial patients. The IDMC is independent from the sponsor and funders. The membership, frequency of meetings, activity (including trial conduct and data review) and authority will be covered in the UCL CCTU IDMC terms of reference.

### Interim analyses

No formal interim analysis is planned, but reports concerning patient safety and key efficacy outcomes will be prepared for regular review by the IDMC who may request an interim analysis if a report raises concern. The IDMC will also be asked to review all the assumptions used for the sample size calculation before the end of recruitment.

### Harms

Serious adverse events will be reported in accordance with the guidance from the National Research Ethics Service (NRES, http://www.nres.npsa.nhs.uk) which is a subdivision of the National Patient Safety Agency http://www.npsa.nhs.uk and Good Clinical Practice (GCP).

Expected intraoperative complications for both arms include all those common to phacoemulsification cataract surgery.^3^ These include anterior capsule tear, posterior capsule tear with or without vitreous loss, choroidal effusion/haemorrhage, IOL exchanged following insertion, zonular dialysis, intraoperative pupil constriction needing intervention and dropped lens fragments or nucleus. Expected intraoperative complications specifically for the laser-assisted arm are failure to dock to laser, aborted or incomplete laser delivery, incomplete capsulotomy identified in surgery requiring manual completion and laser delivery to an inappropriate structure of eye.

Expected postoperative complications for both arms are postoperative uveitis, endophthalmitis, macular oedema, retinal tear or retinal detachment, elevated intraocular pressure requiring treatment, medication allergy or intolerance, corneal oedema, vitreous to wound, other ocular surgery.

Details of any of the complications listed above will be recorded on the case report forms and reported to the Chief Investigator and the IDMC.

### Auditing

Monitoring of this trial will ensure compliance with GCP.

## Ethics and dissemination

### Ethical and safety considerations

The study will be conducted in accordance with GCP guidelines and has received a favourable ethical opinion from the NRES Committee London—City Road and Hampstead (reference number: 14/LO/1937). Trial investigators will ensure that the study (including any approved amendments) is conducted in accordance with the principles of the Declaration of Helsinki. All protocol modifications will be disseminated to all relevant parties.

### Dissemination plan

The results of the trial will be disseminated regardless of the direction of effect. The trial will be reported in accordance with the relevant CONSORT guidance.

Trial findings will be disseminated to all potential beneficiaries of the research including patients, carers and relatives and also doctors, advisory bodies and healthcare Commissioners. This will take the form of papers in high-impact open-access medical journals and also presentations at national and international medical conferences. Trial results will also be disseminated to the trial patients in a one-page summary written in lay language.

### Authorship

Publications generated from the trial will be attributed to the FACT TMG, which will consist of all those who have wholeheartedly collaborated in the trial. The main report will be drafted by the TMG, and the final version will be reviewed by the TSC before submission for publication. TMG members will be named and their affiliations listed in the main report. All publications will be in compliance with the CCTU Publication Policy.

## Footnotes

i Contraindication to use of the AMO Catalys cataract surgery laser, from operator manual version DC-03013-01, Rev B, 02/2014.
